# Muscle mass loss measured with portable ultrasound in hospitalized older adults: The ECOSARC study

**DOI:** 10.1016/j.jnha.2023.100010

**Published:** 2023-12-14

**Authors:** Esther López Jiménez, Marta Neira Álvarez, Rocío Menéndez Colino, Marta Checa López, Concha Grau Jiménez, Patricia Pérez Rodríguez, Brian Vasquez Brolen, Estefanía Arias Muñana, Raquel Ramírez Martín, Cristina Alonso Bouzón, María Solange Amor Andrés, Cristina Bermejo Boixareu, Fátima Brañas, María Alcantud Ibáñez, Rubén Alcantud Córcoles, Elisa Belén Cortés Zamora, Elena Gómez Jiménez, Luis Romero Rizos, Almudena Avendaño Céspedes, Carmen Rosa Hernández Socorro, Pedro Abizanda

**Affiliations:** a Complejo Hospitalario Universitario de Albacete, Albacete, Spain; b Hospital Universitario Infanta Leonor, Madrid, Spain; c Hospital Universitario La Paz, Madrid, Spain; d Hospital Universitario de Getafe, Getafe, Madrid, Spain; e Hospital Virgen del Valle, Toledo, Spain; f Hospital Universitario Puerta de Hierro, Majadahonda, Madrid, Spain; g Hospital Universitario Infanta Sofía, San Sebastián de los Reyes, Madrid, Spain; h Centro de Investigación Biomédica en Red de Fragilidad y Envejecimiento Saludable (CIBERFES), Instituto de Salud Carlos III, Madrid, Spain; i Fundación Hospital Nacional de Parapléjicos, Toledo, Spain; j Facultad de Medicina de Albacete, Universidad de Castilla-La Mancha, Spain; k Facultad de Enfermería de Albacete, Universidad de Castilla-La Mancha, Albacete, Spain; l Hospital Universitario de Gran Canaria, Doctor Negrín, Las Palmas de Gran Canaria, Spain

**Keywords:** Sarcopenia, Ultrasound, Hospitalization, Older adults, Quadriceps rectus femoris

## Abstract

**Objectives:**

The main objective was to analyze the evolution of muscle of the Quadriceps Rectus Femoris (QRF) between admission and discharge, in older adults hospitalized with an acute medical disease in Acute Geriatric Units (AGUs).

**Design:**

Prospective multicentric observational cohort study.

**Setting:**

Seven AGUs from University Hospitals in Spain.

**Participants:**

Hospitalized adults ≥ 70 years old, able to ambulate and without severe dementia.

**Measurements:**

Ultrasound measurements of QRF were acquired at 2/3 distal between anterior-superior iliac spine and patella in both legs by trained Geriatricians. Ultrasound Chison model ECO2 was used. QRF area, thickness, edema, echogenicity, and fasciculations were measured.

**Results:**

From the complete sample (n = 143), in 45 (31.5%) participants, ultrasound images were classified as non-valid by an expert radiologist. Mean age was 87.8 (SD 5.4). Mean hospital stay 7.6 days (SD 4.3). From those with valid images, 36 (49.3%), 2 (2.7%), and 35 (47.9%) presented a decrease, equal values, or an increase in QRF area from baseline to discharge, respectively, and 37 (50.0%), 2 (2.7%), and 35 (47.3%) presented a decrease, equal values, or an increase in QRF thickness, respectively. 26 (35.6%) presented a decrease in more than 0.2 cm^2^ of QRF area, and 23 (31.1%) a decrease in more than 0.1 cm of QRF thickness. Only 4 (5.4%) patients presented new edema, while 13 (17.6%) worsened echogenicity.

**Conclusion:**

One third of older adults develop significant muscle loss during a hospitalization for acute medical diseases.

**Trial registration number:**

NCT05113758

## 1 Introduction

Sarcopenia is highly prevalent in hospitalized older adults, between 10.2%–81.4% depending on the definition, the methodology, and the setting [[1], [2], [3]], with a pooled prevalence of 37% (95% confidence interval 26%–48%) [4]. Sarcopenia is associated transversally with diseases like hip fractures, vertebral fractures, malnutrition, chronic kidney disease, stroke, COVID-19, and also with comorbidity or admission to critical care and surgery units [[5], [6], [7]]. In addition, sarcopenia in older inpatients is associated with health adverse outcomes like disability in activities of daily living (ADL), mobility loss, falls, fractures, infections, hospital readmissions, in-hospital complications, post-operative hospital stay, post-COVID hospital stay, post-operative complications and outcomes, institutionalization, hospitalization costs, and mortality [5,[7], [8], [9], [10], [11], [12], [13], [14], [15]]. However, other studies in hospitalized older adults have not found a clear relationship between sarcopenia and mortality [16].

There is a great heterogeneity in the results regarding the association between sarcopenia or muscle quantity/quality with health outcomes in hospitalized older adults for several reasons. First, studies have been conducted in different settings with different populations, including acute geriatric units (AGUs), post-acute geriatric units, geriatric psychiatry units, general medicine departments, neurology departments, COVID-19 units, rehabilitation units, day hospital, critical care units, or surgery departments [[1], [2], [3],^5^,^6^,^9^,^10^,^12^,^13^,[16], [17], [18], [19], [20], [21], [22], [23], [24]]. Second, different methods for muscle quantity/quality have been used, including dual energy x-ray absorptiometry (DXA), bioelectrical impedance analysis (BIA), anthropometric measurements, screening scales, computed tomography (CT) scans, or ultrasound imaging [1,3,[5], [6], [7],[9], [10], [11], [12],[17], [18], [19], [20], [21], [22], [23], [24]]. Third, different criteria have been used [1], from the first European Working Group on Sarcopenia in Older People (EWGSOP) to the second EWGSOP-2 criteria, the Asian Working Group for Sarcopenia (AWGS) recommendations, the Foundation for the National Institutes of Health (FNIH) criteria, the International Working Group on Sarcopenia (IWGS), the Special Interest Group of Sarcopenia, Cachexia and Wasting Disorders (SIG), or mixed criteria [1,2,5,7,10,16,18]. Finally, imaging diagnostic techniques like DXA, BIA, CT or magnetic resonance imaging (MRI), functional tests like gait speed or the Short Physical Performance Battery (SPPB), strength and power measurements, or screening scales like SARC-F may be difficult to acquire in hospitalized older adults.

Ultrasonography is a portable technique, non-invasive, radiation-free, and cost-effective, able to determine not only quantitative muscle measures, but also qualitative ones, including mechanical properties, echogenicity (fat infiltration, fibrosis, myonecrosis), and microcirculation. Recently, ultrasonography has been approved as a valid and reliable imaging method for the assessment of skeletal muscle mass [25,26], more easy-to-use and suitable for hospitalized older adults than other techniques [17]. However, there is an urgent need for a standardization of the measurement technique, the identification of best muscles for image acquisition, cut-off values in different conditions, and longitudinal information of muscle data [19,25,27]. In addition, reliability and validity values from ultrasound studies have been obtained under strictly controlled conditions, which are likely to decrease in real clinical practice [15,28].

For these reasons, we designed and conducted the ECOSARC Project [29], with the main objective of longitudinally estimate, by means of portable ultrasound under real-life conditions, parameters of muscle quantity and quality (anterior rectus femoris muscle area, thickness and echostructure) in older adults hospitalized in AGUs for medical reasons. In this manuscript we present the first descriptive results of the project.

## 2 Methods

The ECOSARC Project is a prospective, observational, multicenter cohort study in older adults admitted to Acute Geriatric Units (AGU) of Spain for acute medical reasons. The complete protocol, rationale, design, and methodology have been published elsewhere [29]. Trial registration number NCT05113758.

In brief, the ECOSARC project included older adults (age ≥ 70 years) hospitalized by medical diseases in AGUs of 7 University Hospitals of Spain from May 2019 to January 2022. Other inclusion criteria were the ability to walk with or without help, previous to the admission, and informed consent signed by the patient or legal representative. Exclusion criteria were terminal conditions or life expectancy of less than 6 months, impossibility or refusal to undergo muscle ultrasound, refusal of follow-up, severe dementia, and/or impossibility in the opinion of the investigators to complete the necessary data for the study.

Baseline and discharge clinical data were collected from patients and caregivers in person by a researcher and from the patients’ clinical records. Demographic characteristics, main diagnosis on admission, number of drugs usually consumed, body mass index (BMI), Holden Functional Ambulation Classification (FAC), Barthel index of basic activities of daily living (BADL), Charlson Comorbidity Index, Global Deterioration Scale from Reisberg (GDS), FRAIL instrument, SARC-F instrument, SPPB, hand grip strength (kg) using a digital JAMAR dynamometer, Mini Nutritional Assessment Tool - Short Form Questionnaire (MNA-SF®), and days of hospital stay were collected. Description and references for all these instruments can be found elsewhere [29].

### 2.1 Muscle ultrasound measurements

Ultrasound measurements were collected by 14 geriatricians (two at each site) who received a 4 h on-site training by an expert radiologist (CRHS). To avoid uncorrect imaging acquisition and analysis during the study, all the images were supervised by the expert radiologist. This expert first discarded wrong images, and second, made corrections to the correct images regarding any of the data retrieved by the geriatricians.

**Fig. 1:**
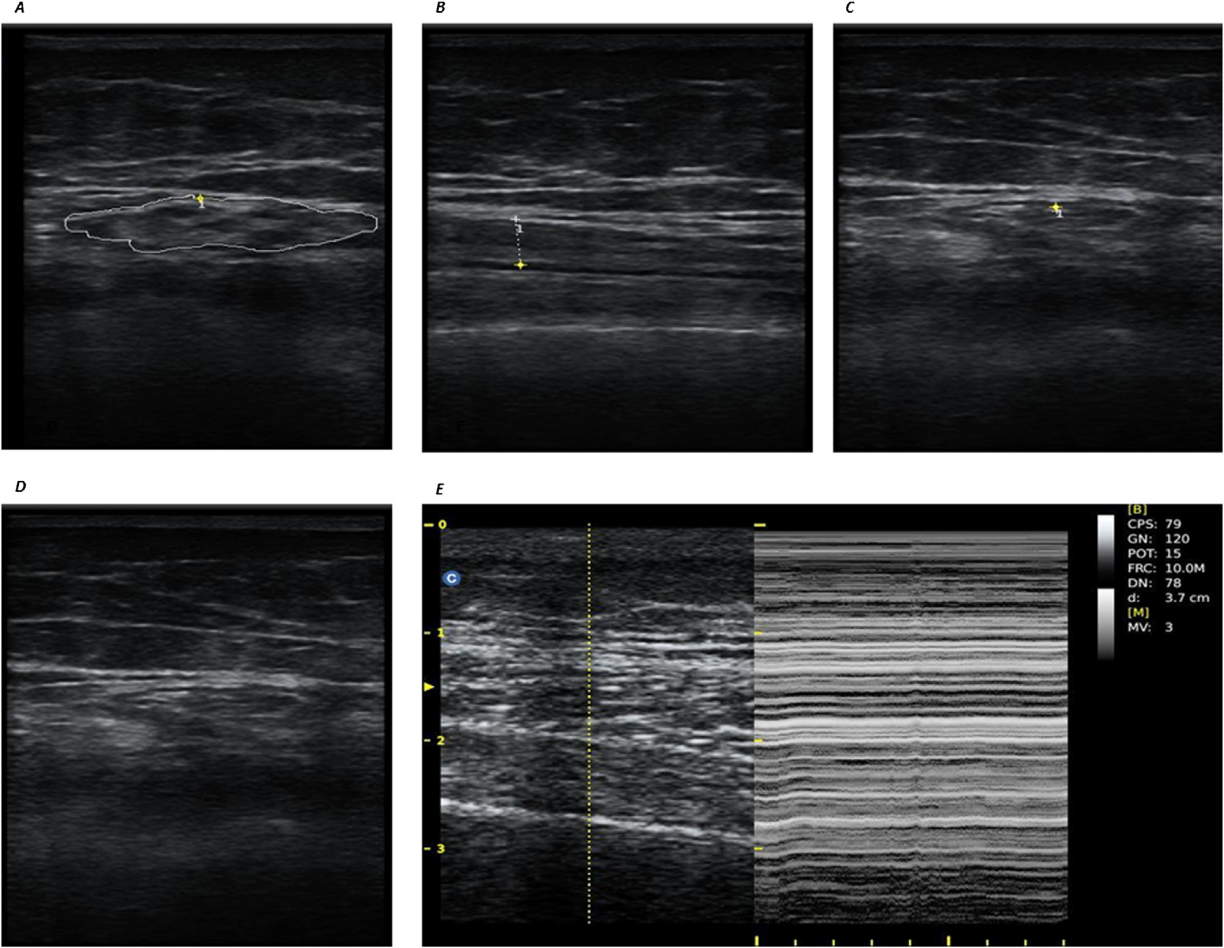
Measurements at two thirds of the lower thigh. *A: Cross-sectional area of Quadriceps rectus femoris, transversal view (mode b). B: Muscle thicknes in longitudinal view (mode b) of Quadriceps rectus femoris. C: tendon thicknes in transversal view (mode b). D: Edema and echogenicity (in the image, presence of edema and echogenicity grade 3 compatible with fat infiltration). E: Fasciculations.*

Muscle ultrasound measurements were acquired baseline in the first 24 h after admission, and the day of discharge, using a protocol previously validated [29], using a Chison model ECO2 ultrasound system (Chison Medical Technologies, Co. Ltd, Wimxu District Wuxi, Jiangsu, China) and a multifrequency linear-array probe (width of probe 38−58 mm). Patients had to lay down in supine position in the bed, with knees extended and relaxed to full extension. The probe had to be aligned perpendicularly to the longitudinal and transversal axes of Quadriceps Rectus Femoris (QRF), for transverse and longitudinal images acquisition. The probe was situated two-thirds of the way along the femur length, measured between the upper pole of the patella and the anterior superior iliac spine. Measurements were collected from both legs individually, and the mean value between both legs was calculated for every parameter. They included the cross-sectional area (CSA) of the QRF (mode b, in cm^2^), the muscle thickness in longitudinal view (mode b, in cm), the intramuscular central tendon thickness in mm with an insonation angle perpendicular to the tendon, the echogenicity of the muscle (1. normal; 2. heterogeneous; 3. fat infiltration; 4. atrophy due to fasciitis and necrosis), the presence or absence of edema in the subcutaneous cellular tissue, the intramuscular and intrafascial fluid, and finally the presence or absence of muscle fasciculations using video testing (Fig. 1) [30].

### 2.2 Statistical methods

Descriptive data are presented using means and standard deviations (SD), and number and percentage of participants, as needed. Kolmogorov-Smirnov test was used to determine data distributions. Variations between hospital admission and discharge ultrasound values, and clinical data, were calculated using paired *t*-tests, or Mc-Nemar chi-square tests when necessary. Stratification by sex or main diagnosis on admission was analyzed with *t*-test and ANOVA test using posthoc Bonferroni analyses. The distribution of QRF CSA and thickness differences between admission and discharge were determined and presented in graphics. A decrease in more than 0.2 cm^2^ of QRF CSA, and in more than 0.1 cm of QRF thickness were considered relevant and not produced by intra-observer variation as previously described [22]. Percentage of participants above these cut-points is described. Finally, comparisons between qualitative ultrasound measurements and QRF CSA and thickness were determined using *t*-test analyses. All analyses were performed using SPSS (Statistical Package for Social Sciences, version 24.0. Armonk, NY: IBM Corp).

### 2.3 Ethics

The study protocol was approved by the Ethics Review Committee of Albacete (“Comité de Ética en Investigación con medicamentos de Albacete”), record 01/09/2019). In accordance with the Declaration of Helsinki and its subsequent amendments, every patient will have the right to leave the study at any time, for any reason, without having to give explanations and without repercussions on his or her aftercare. Likewise, the investigating physician will have the right to withdraw a patient from a study when he/she believes it is in the patient's best interest. Since this is a registry, exceptionally, a withdrawal criterion could be applied if, during data collection at the single visit, the patient or his/her representative decides to reconsider his/her participation in the study. All the information obtained from the study participants will be treated confidentially, complying with the Organic Law 3/2018 on Personal Data Protection with its last update on July 25, 2019.

## 3 Results

From the complete sample (n = 143), in 45 (31.5%) participants, ultrasound images or measurements were classified as non-valid by the expert radiologist. Patients included per each site with the number and percentage of non-valid ultrasound measurements were 0/23 (0.0%), 4/30 (13.3%), 6/33 (18.2%), 5/26 (19.2%), 12/13 (92.3%), 9/9 (100%) and 9/9 (100%), respectively. In addition, from the patients with valid ultrasound assessments, in 28 cases there was a slight discrepancy in echogenicity, in 2 cases in edema, and in 15 cases in fasciculations assessment between the expert radiologist and the geriatricians. Table 1 presents the baseline characteristics of participants with valid and non-valid ultrasound measurements. Only differences in frailty and ambulation on admission were detected between those with valid and non-valid measurements.

**Table 1:** Baseline characteristics of patients with valid and non-valid ultrasound measurements.

	Non-valid ultrasound (n = 45)		Valid ultrasound (n = 98)		*p*
	n	Mean (SD) n (%)	n	Mean (SD) n (%)	
Age	45	86.3 (5.7)	98	87.8 (5.4)	.121
Female sex	45	22 (48.9)	98	61 (62.2)	.133
Institutionalized	44	10 (22.7)	98	15 (15.3)	.283
Main diagnosis on admission	45		98		.202
Heart failure/CAD		8 (17.8)		31 (31.6)	
Pulmonary infection		9 (20.0)		19 (19.4)	
Urinary infection		28 (62.2)		48 (49.0)	
BMI (kg/m^2^)	43	28.1 (5.6)	93	27.2 (5.4)	.400
Barthel index 15 d before admission	42	82 (18)	98	83 (17)	.542
Barthel index on admission	38	62 (28)	98	59 (26)	.506
FRAIL 15 d before admission	45	1.7 (1.3)	98	1.7 (1.2)	.944
FRAIL on admission	45	1.6 (1.5)	98	2.2 (1.1)	.007
SPPB on admission	45	4.1 (4.0)	92	3.1 (2.7)	.117
SARC-F 15 d before admission	45	4.0 (2.9)	96	3.6 (2.5)	.455
FAC Holden on admission	43	3.4 (1.3)	98	3.9 (1.1)	.016
Hand grip strength (kg)	44	16.4 (9.5)	96	14.7 (9.6)	.331
Charlson index	45	2.9 (2.3)	98	2.5 (2.0)	.314
Number of drugs before admission	45	8.9 (3.5)	95	8.9 (3.5)	.964
MNA-SF	44	9.4 (2.9)	95	9.6 (2.3)	.729
Global Deterioration Scale Reisberg	45	2.4 (1.5)	98	2.1 (1.4)	.267
Days of hospitalization	42	8.2 (10.0)	92	7.6 (4.3)	.637

BMI: Body mass index; d: days; SPPB: Short Physical Performance Battery; FAC: Functional Ambulation Classification; MNA-SF; Mini-Nutritional Assessment Short Form; CAD: Coronary artery disease.

Table 2 presents the baseline muscle ultrasound values of patients with valid and non-valid ultrasound measurements. Differences were detected mainly in QRF CSA, in tendon thickness, and in fasciculations assessment, indicating the most frequent technique errors realized by the physicians.

**Table 2:** Baseline muscle ultrasound values of patients with valid and non-valid ultrasound measurements.

	Non-valid ultrasound			Valid ultrasound			*p*
	n	Mean (SD) n (%)	Range	n	Mean (SD	Range	
QRF area RL (cm^2^)	34	2.44 (1.02)	0.70−4.67	95	1.80 (0.74)	0.19−4.08	.002
QRF area LL (cm^2^)	32	2.29 (0.92)	0.80−4.26	95	1.84 (0.77)	0.70−4.29	.009
QRF area BL (cm^2^)	32	2.35 (0.91)	0.84−4.20	95	1.82 (0.68)	0.69−3.65	.004
QRF thickness RL (cm)	35	0.86 (0.42)	0.36−2.28	94	0.76 (0.27)	0.07−1.68	.108
QRF thickness LL (cm)	34	0.82 (0.37)	0.30−1.84	94	0.74 (0.28)	0.10−1.83	.171
QRF thickness BL (cm)	34	0.83 (0.35)	0.33−2.06	94	0.75 (2.25)	0.09−1.46	.176
Tendon thickness RL (mm)	33	0.47 (0.53)	0.06−1.70	94	0.95 (0.41)	0.40−2.10	.000
Tendon thickness LL (mm)	33	0.66 (1.27)	0.05−7.00	93	0.92 (0.40)	0.30−2.60	.242
Tendon thickness BL (mm)	31	0.46 (0.53)	0.06−1.75	93	0.93 (0.37)	0.40−2.20	.000
Edema RL (yes)	37	13 (35.1)	–	95	48 (50.5)	–	.111
Edema LL (yes)	36	14 (38.9)	–	93	47 (40.5)	–	.235
Edema BL (yes any)	36	14 (38.9)	–	94	48 (51.1)	–	.214
Echogenicity RL	39		–	96		–	.214
Normal		1 (2.6)			14 (14.6)		
Heterogeneous		13 (33.3)			27 (28.1)		
Fat infiltration		23 (59.0)			48 (50.0)		
Atrophy		2 (5.1)			7 (7.3)		
Echogenicity LL	38		–	95		–	.223
Normal		1 (2.6)			14 (14.7)		
Heterogeneous		11 (28.9)			27 (28.4)		
Fat infiltration		24 (63.2)			48 (50.5)		
Atrophy		2 (5.3)			6 (6.3)		
Worst echogenicity BL	39		–	96		–	.153
Normal		1 (2.6)			14 (14.6)		
Heterogeneous		10 (25.6)			27 (28.1)		
Fat infiltration		26 (66.7)			48 (50.0)		
Atrophy		2 (5.1)			7 (7.3)		
Fasciculations RL (yes)	40	23 (57.5)	–	96	92 (95.8)	–	.000
Fasciculations LL (yes)	39	23 (59.0)	–	96	93 (96.9)	–	.000
Fasciculations BL yes any	40	24 (60.0)	–	96	93 (96.9)	–	.000
Fasciculations BL no any	39	17 (43.6)	–	96	4 (4.2)	–	.000

QRF: Quadriceps rectus femoris; RL: Right leg; LL: left leg; BL: Mean of both legs.

Table 3 presents the differences between baseline and discharge ultrasound measurements for participants with valid ultrasound measurements. Muscle values decreased from baseline to discharge for all the measurements, reaching only statistical significance for QRF thickness with a loss of muscle thickness of 0.04 cm (95% CI 0.00 to 0.08; p = 0.043). Regarding edema, echogenicity, and fasciculations, we could not find differences between baseline and discharge assessments, although there was a small tendency towards worse echogenicity.

**Table 3:** Differences between baseline and discharge ultrasound measurements for participants with valid ultrasound measurements.

		Baseline	Discharge	Mean difference 95% CI	*p*
	n	Mean (SD) n (%)	Mean (SD) n (%)		
QRF area RL (cm^2^)	74	1.83 (0.70)	1.71 (0.68)	0.12 (-0.06 to 0.31)	.177
QRF area LL (cm^2^)	74	1.92 (0.82)	1.79 (0.66)	0.13 (-0.05 to 0.32)	.156
QRF area BL (cm^2^)	74	1.88 (0.69)	1.75 (0.60)	0.13 (-0.03 to 0.29)	.099
QRF thickness RL (cm)	74	0.79 (0.25)	0.74 (0.24)	0.04 (-0.01 to 0.09)	.079
QRF thickness LL (cm)	74	0.77 (0.28)	0.73 (0.25)	0.04 (-0.02 to 0.09)	.188
QRF thickness BL (cm)	74	0.78 (0.24)	0.74 (0.24)	0.04 (0.00 to 0.08)	.043
Tendon thickness RL (mm)	70	0.92 (0.40)	0.87 (0.37)	0.05 (-0.04 to 1.58)	.257
Tendon thickness LL (mm)	71	0.91 (0.42)	0.83 (0.37)	0.07 (-0.04 to 1.83)	.189
Tendon thickness BL (mm)	69	0.91 (0.39)	0.85 (0.33)	0.06 (-0.03 to 0.15)	.187
Edema RL (yes)	74	35 (47.3)	31 (41.9)	–	.289
Edema LL (yes)	71	34 (47.9)	32 (45.1)	–	.727
Edema BL (yes any)	73	35 (47.9)	34 (46.6)	–	1.00
Echogenicity RL	74				.596
Normal		12 (16.2)	11 (14.9)	–	
Heterogeneous		22 (29.7)	22 (29.7)	–	
Fat infiltration		36 (48.6)	34 (45.9)	–	
Atrophy		4 (5.4)	7 (9.5)	–	
Echogenicity LL	74				.391
Normal		12 (16.2)	11 (14.9)	–	
Heterogeneous		23 (31.1)	21 (28.4)	–	
Fat infiltration		36 (48.6)	34 (45.9)	–	
Atrophy		3 (4.1)	8 (10.8)	–	
Worst echogenicity BL	74				.469
Normal		12 (16.2)	11 (14.9)	–	
Heterogeneous		22 (29.7)	21 (28.4)	–	
Fat infiltration		36 (48.6)	34 (45.9)	–	
Atrophy		4 (5.4)	8 (10.8)	–	
Fasciculations RL (yes)	73	71 (97.3)	72 (98.6)	–	1.00
Fasciculations LL (yes)	73	71 (97.3)	72 (98.6)	–	1.00

QRF: Quadriceps rectus femoris; RL: Right leg; LL: left leg; BL: Mean of both legs.

**Fig. 2:**
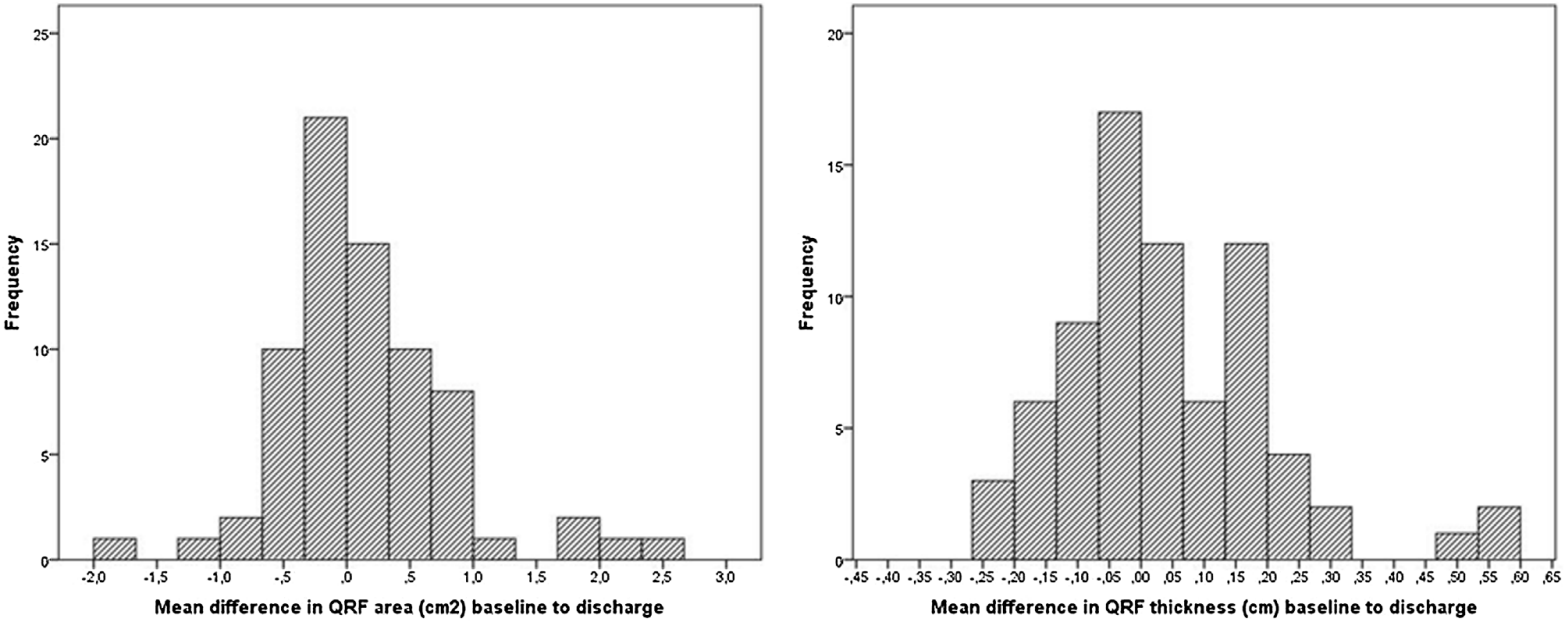
Histograms of differences in QRF measurements from baseline to discharge.

Fig. 2 presents the distribution of QRF CSA and thickness (mean of both legs) changes between baseline and discharge assessments. From the participants with valid ultrasound data, 36 (49.3%), 2 (2.7%), and 35 (47.9%) presented a decrease, equal values, or an increase in QRF CSA from baseline to discharge, respectively, and 37 (50.0%), 2 (2.7%), and 35 (47.3%) presented a decrease, equal values, or an increase in QRF thickness from baseline to discharge, respectively. 26 (35.6%) participants presented a decrease in more than 0.2 cm^2^ of QRF CSA, and 23 (31.1%) a decrease in more than 0.1 cm of QRF thickness. However, we were not able to find associations between changes in ultrasound measurements from admission to discharge, and changes in physical function (SPPB), hand grip strength, or disability in BADL (Barthel index). Morover, stratification by sex or by main diagnosis on admission did not retrieve differences either in ultrasound parameters or in functional tests.

Only 4 (5.4%) patients presented new edema in ultrasound assessments between admission and discharge, while 13 (17.6%) worsened echogenicity. Participants who worsened echogenicity presented a higher non-significant loss of QRF CSA (0.43 cm^2^ vs 0.07 cm^2^; *p* = 0.083), and thickness (0.10 cm vs 0.03 cm; *p* = 0.378) respectively. Furthermore, participants with a significant improvement (>0.1 cm) in QRF thickness had a higher prevalence of better echogenicity (level 1, normal) than those without change (>−0.1 cm to ≤0.1 cm), or than those with a significant worsening (<−0.1 cm), both on admission 33.3% - 16.7% - 4.3% and at discharge 20.1% - 16.7% - 8.7% respectively.

## 4 Discussion

To our knowledge, this is the first multicentric study conducted by geriatricians in AGUs trying to identify muscle loss during a hospitalization for an acute medical disease, and the main result is that one third of older adults develop significant muscle loss during a hospitalization for acute medical diseases using ultrasound measurements, with a mean QRF thickness loss of 6.4%. In addition, this muscle loss takes place with a short mean length of stay, 7.6 (SD 4.3) days. This muscle loss has also been described in patients with acute exacerbations of chronic respiratory disease, with a drop in Quadriceps thickness by 8.3% during the period of hospitalisation [19]. Factors like hospital-associated immobility [31], chronic inflammation, anemia, body composition, anorexia, or malnutrition [32] have been described for this finding.

Quantitative muscle results are not in disagreement with reference values in other populations. In a critical care unit study using our same methodology, authors described ultrasound measurements in patients with clinical neuromuscular acquired weakness (mean age 62 years) and healthy controls (mean age 60 years); they found QRF thickness median value of 0.57 cm and 1.14 cm respectively, and QRF CSA median value of 1.0 cm^2^ and 3.6 cm^2^ respectively [29]. Our data, with a QRF thickness mean value of 0.75 cm, and a QRF CSA mean value of 1.82 cm^2^, are situated halfway between those healthy controls and critical care patients, indicating that older medical inpatients may be a population with intermediate muscular values between healthy adults and critical care patients. In another study, Guerreiro et al. presented median values of QRF in a population of older inpatients, in Brazil. In a sample slightly younger than ours, 78 years, but with similar function and comorbidity, they describe median values of 1.65 cm for both QRF and vastus intermedius [33]. Authors do not present median values for QRF, but in the image included in the manuscript, 0.53 cm is described, close to our figures. Other studies in younger populations have described higher values of QRF thickness. A study in 30 middle-aged to older males and females with mean age 59.9 years described an ultrasound mean QRF CSA of 4.6 cm^2^ and thickness of 1.5 cm [34], three-fold and two-fold higher values respectively than in our population. Finally, another study on 95 healthy volunteers, 47males, with ages ranging from 17 to 90 years, showed a mean QRF thickness of 4.12 cm for men and 3.61 cm for women, although measurements were done in the mid muscle [35].

Although our results showed that half of the participants presented a decrease in QRF thickness and CSA during the hospitalization period, we decided to eliminate those results that have been associated with inter-rater bias in previous studies, 0.1 cm for thickness and 0.2 cm^2^ for cross-sectional area, based on Bland-Altman analysis. This approach yielded a significative decrease in muscle mass approximately in one third of participants [22]. However we were surprised that almost half of our sample increased muscle measurements during hospitalization, an unlikely finding in an AGU with very vulnerable older adults and a short stay. Bivariate analysis showed that only a better echogenicity, both on admission and at discharge, was associated with an increase in muscle thickness, suggesting lower fat infiltration and necrosis, and a “better muscle quality”. Neither associations with edema nor changes in echogenicity were related to increases in muscle mass. Probably a better muscle quality baseline may have produced a higher muscle recovery after and acute episode. However, other confounding factors that could interfere with changes in muscle ultrasound values like days of immobility, rehabilitation, nutritional aspects like diet, calorie or protein intake, comorbidity, catabolic status, or medications like glucocorticoids were not adequately assessed and should promote further research [36].

From a qualitative point of view, half of our sample presented muscle edema and fat infiltration echogenicity on admission, without significant changes after discharge. Echo-intensity has been described as a useful parameter to predict hospital-related complications in acute hospitalized older adults [12]. Moreover, intramuscular adipose tissue of the quadriceps has been more strongly related to declines in ADL than loss of muscle mass [20], muscle mass and echo-intensity are close related [21]. The extent to what edema and fat infiltration may lead to an overestimation of skeletal muscle area is not clear, because both aspects affect muscle attenuation, and also, the separate effects of these on muscle quality are difficult to distinguish [37].

The quantification of muscle mass and muscle quality during hospitalization may be of relevance not only to be used as a marker of poor outcomes, but also to identify categories of older patients at risk for functional decline. In these patients, an individualized multicomponent exercise training program, with special emphasis on resistance exercises and muscle power training, has demonstrated to improve physical function, maximal muscle strength, and muscle power [38].

In our study, a global overview of almost all the changes in ultrasound measurements during hospitalization shows rather insignificant differences with confusing directionality. For this reason, the clinical significance of our findings is not clear, moreover after describing that changes in muscle measurements are not associated with changes in physical function or disability. These findings may appear to be in contradiction with previous ones in intensive care patients, showing that low skeletal muscle measurements influence clinical outcomes [23]. However, ICU patients are not similar to older adults, and a dissociation between muscle mass and physical performance has been previously described in older adults populations [39]. Another possible explanation is that we may need better ultrasound biomarkers other than thickness and CSA, to detect changes in old, senescent, and damaged muscles of older adults. Quantitative muscle assessment (QUS) [40] or measurements with the muscle in movement [41], similar to the ejection fraction in the heart, may show changes in the physiologic functions of the muscle leading to changes in physical performance.

The main limitation of the study is that geriatricians were not able to acquire valid images in almost one third of the patients (31.5%), and also in one-out-of-four of the valid ones there were slight measurement errors, after careful review by an external expert radiologist. This variability was present although specific training was provided. The rate of non-valid images varied greatly between sites, from 0 to 100%, and was highly dependent on the previous ultrasound level of expertise of the operators. Operator-related variability of echography assessment has been described in multiple anatomic regions, pathologies, populations, and settings [42], although training programs have demonstrated to improve the level of intra- and inter-rater agreement [43]. Regarding skeletal muscle ultrasound assessment, the experience and skill levels of both sonographers and those analyzing the image offline can influence the reliability and validity of the quantitative and qualitative measurements [44]. Operator-related factors such as probe orientation and skin compression are some, but not all, of the factors influencing accuracy of image acquisition [44,45]. Moreover, in older adults with acute diseases, frailty, or disability, patient-related factors like muscle oedema, necrosis or atrophy, malnutrition, obesity, or lack of patient collaboration may also be of relevance [22,25]. However, from our results, we are able to propose that a better training and a well-defined examination protocol should be offered to geriatricians and other clinicians without previous expertise in ultrasound muscle assessment when analyzing skeletal muscle characteristics in older adult populations, and that standardized point-of-care ultrasound imaging programs are needed in Geriatric Medicine [23,25].

Other limitations are an unknown feasibility of the selected protocol for its use in hospitalized older adults instead of critical care patients, and the heterogeneity of the sample regarding pathologies, functional status or medicines. However, we think that the results obtained will be of great interest to the scientific geriatric community to assess the validity of ultrasound measurements for the detection and follow-up of sarcopenia in hospitalized older adults. The strengths of the study are the multicentric and longitudinal design, the use of a previous validated protocol, the external validation from an expert radiologist, and the previous training of the geriatricians. In addition, ultrasonography has been shown as a reliable and valid diagnostic method for the quantitative assessment of appendicular muscle mass in sarcopenia in older people. The thickness and CSA of the QRF seem to be proper ultrasound parameters to predict muscle mass in sarcopenia [46].

Measurement of muscle mass and sarcopenia is a challenge in hospitalized older adults. Older adults in AGUs are a vulnerable population in a complex clinical environment, with high rates of frailty, disability, geriatric syndromes, multimorbidity, and polypharmacy. In addition, chronic muscle changes are very frequent in this population [22]. For all these reasons, bed-side assessments of imaging measurements, functional testing or questionnaires may be difficult to be correctly acquired [8]. Protocols should be carefully adapted and designed to optimize data validity and reliability, reducing drop-outs from studies.

It is not clear if present recommended measurements of muscle quantity like area or thickness are the best ones to identify sarcopenia, because they only determine static volume parameters, but not muscle movement or function. The use of contemporary artificial intelligence-assisted software to more comprehensively characterize changes in muscle quality, and the integration of cutting-edge computational analytic techniques to quantify both quantitative and qualitative muscular changes, in both a static and dynamic acquisition procedure, could better identify sarcopenia, loss of muscle function, and delineate risks for adverse post-discharge outcomes. Although methodology improvement is needed, ultrasound imaging may be a good tool for increasing the successful rates of sarcopenia assessment in this population [8,18], and although some assessment protocols have been defined, consensus is still lacking [23,47]. Finally, ultrasonography provides the opportunity to measure the muscle in movement, not available by other techniques like BIA, DXA, MRI or CT scan [48].

## Funding

This study was supported by Abbott Nutrition Iberia, and also by CIBERFES (CB16/10/00408), Instituto de Salud Carlos III, Ministerio de Economía y Competitividad, España. Ayuda cofinanciada por el Fondo Europeo de Desarrollo Regional FEDER Una Manera de hacer Europa.

## Authors' contributions

PA is coordinating investigator ECOSARC study and developed the study desing. EEJ, MNA, RRM, CAB, MSAA, CBB, FB, RMC, EAM, MCL, CGJ, PPR, MAI, BVB, RAC and LRR participated in the design of the study, assistant in development protocol. CRSH, JO and LMPL gave expert tips. AAC, EBCZ and EGJ reviewed final writing. All authors read and approved the final manuscript.

## Declaration of interests

The authors declare the following financial interests/personal relationships which may be considered as potential competing interests:

Pedro Abizanda reports financial support was provided by Biomedical Research Network Centre of Fragility and Healthy Aging. Pedro Abizanda reports financial support was provided by Abbott Laboratories. If there are other authors, they declare that they have no known competing financial interests or personal relationships that could have appeared to influence the work reported in this paper.

## Ethics statement

The experiments comply with the current Spanish laws.
